# What is the relationship between the breech presentation and femoral trochlear dysplasia? An experimental study of the breech presentation model in neonatal rats

**DOI:** 10.1186/s12891-022-05023-3

**Published:** 2022-01-17

**Authors:** Weifeng Li, Shengjie Wang, Shiyu Tang, Zhenyue Dong, Fei Wang

**Affiliations:** Department of Joint Surgery, the Third Hospital of Hebei Medical University, 139 Ziqiang Road, Shijiazhuang, 050051 Hebei China

**Keywords:** Trochlear dysplasia, Breech presentation, Animal model;Rat

## Abstract

**Background:**

The relationship between breech presentation and trochlear dysplasia has been confirmed. However, the pathological process of breech-related trochlear dysplasia remains unclear. This study aimed to establish an animal model to simulate breech presentation and to analyze the pathological process of the femoral trochlea.

**Materials and Methods:**

One hundred and twenty neonatal rats were randomly assigned into a control group and two experimental groups that were swaddled (using surgical tape) to keep the hip flexed and knees extended to simulate human breech presentation for the 5 days (short Swaddling) and the 10 days (prolonged Swaddling) of life. Gross and cross-sectional observation, histological staining measurement in two experimental time points (5 and 10 days after birth) were conducted to evaluate the morphological changes of the femoral trochlea.

**Results:**

The incidence of trochlear dysplasia increased with the Swaddling time. Rats in the prolonged Swaddling group had the high prevalence of trochlea dysplasia (52 of 60), followed by short Swaddling group (42 of 60). Gross and cross-sectional observation showed a shallower trochlea groove in two experimental groups. Histologicalstaining measurement indicated that the trochlear sulcus angle and trochlear sulcus depth were significantly different between the experimental group and the control group since day 5 and day 10.

**Conclusion:**

In this model, breech presentation had an adverse effect on neonatal knees and could induce trochlear dysplasia. In addition, this study also showed that the more time in breech presentation, the more incidence of trochlear dysplasia.

## Introduction

Trochlear dysplasia is a spectrum of anatomical morphological abnormalities of the distal femur [1, 2]. Previous studies have shown the high incidence of trochlear dysplasia among young children and teenagers [3, 4]. The risk factors for trochlear dysplasia include mechanical stress, genetic factor, global joint hypermobility and breech presentation, among which breech presentation is a focal point of research [5–11]. Previous studies demonstrate the relationship between breech presentation and trochlear dysplasia through epidemiological methods [11]. However, few reports have described the mechanism of this correlation. Also, there have been no studies indicating the pathological process of breech-related trochlear dysplasia. Since there has been no accurate method to induce breech presentation in utero, and the trials on human neonates would be controversial, animal experimental model becomes an indispensable choice. Ren has reported the pathogenesis of breech-related developmental dysplasia of the hip based on a newborn rat model [12]. It is known that there is the similar phenomenon of articular remodelling, such as trochlear dysplasia and hip dysplasia in children [13, 14]. Thus, we speculated whether breech presentation could also be a predisposing factor for trochlear dysplasia.

The purpose of the present study was to establish a simulating breech presentation animal model in neonatal rats using the surgical tape and to analyze the pathological development of the trochlear dysplasia.

## Materials and Methods

The study was approved by the Ethics Committee.

**Fig. 1:**
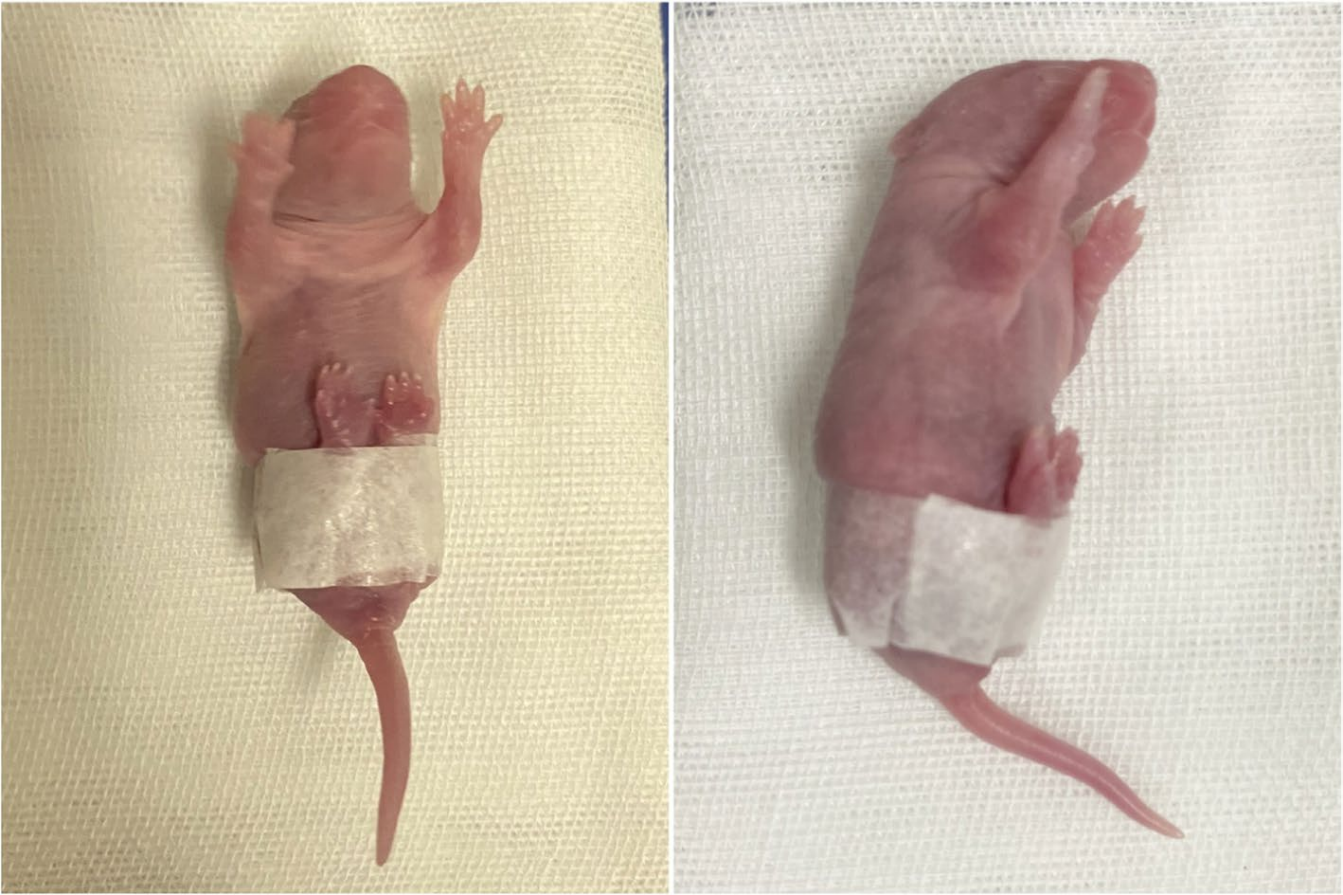
The swaddling model of frank-breech presentation. The rat was swaddled with surgical tape to keep the hip flexed and knees extended so as to simulate human frank-breech presentation

One hundred and twenty neonatal Wistar rats provided by the local Animal Center were assigned into three groups. All the rats were paired sexually. The control group included 120 knee joints, and each experimental group included 60 knee joints. Rats in control group were left untreated. The short swaddling group in which rats were swaddled for 5 days. The prolonged swaddling group rats in this group underwent swaddled for 10 days. Rats in two experimental groups were swaddled with surgical tape (3 MD urapore, St.Paul, Minnesota) to keep the hips flexed and knees extended so as to simulate the human breech presentation (Fig. 1) [12]. The rats were permitted to release from the swaddling for approximately 30 min per day. All rats were fed by their mothers.

### Gross observation

In total, 120 neonatal Wistar rats were euthanized by excessive intraperitoneal injection of pentobarbital sodium (200 mg/kg) at 5 and 10 days after birth respectively [15, 16]. The skin and internal organs were removed carefully and the bone tissues of distal femur were fixed in 4% paraformaldehyde overnight. The anatomic morphology of the femoral trochlea was observed and recorded.

### Cross-sectional observation

In order to observe the cross-sectional femoral trochlea, we chose the slice just the point of proximal to the posterior condyle. From this level, all of the femoral trochlea were sectioned axially using a scalpel [17]. Then, the cross-sectional anatomy of trochlea groove was observed in three groups.

### Haematoxylin and eosin (HE) staining

**Fig. 2:**
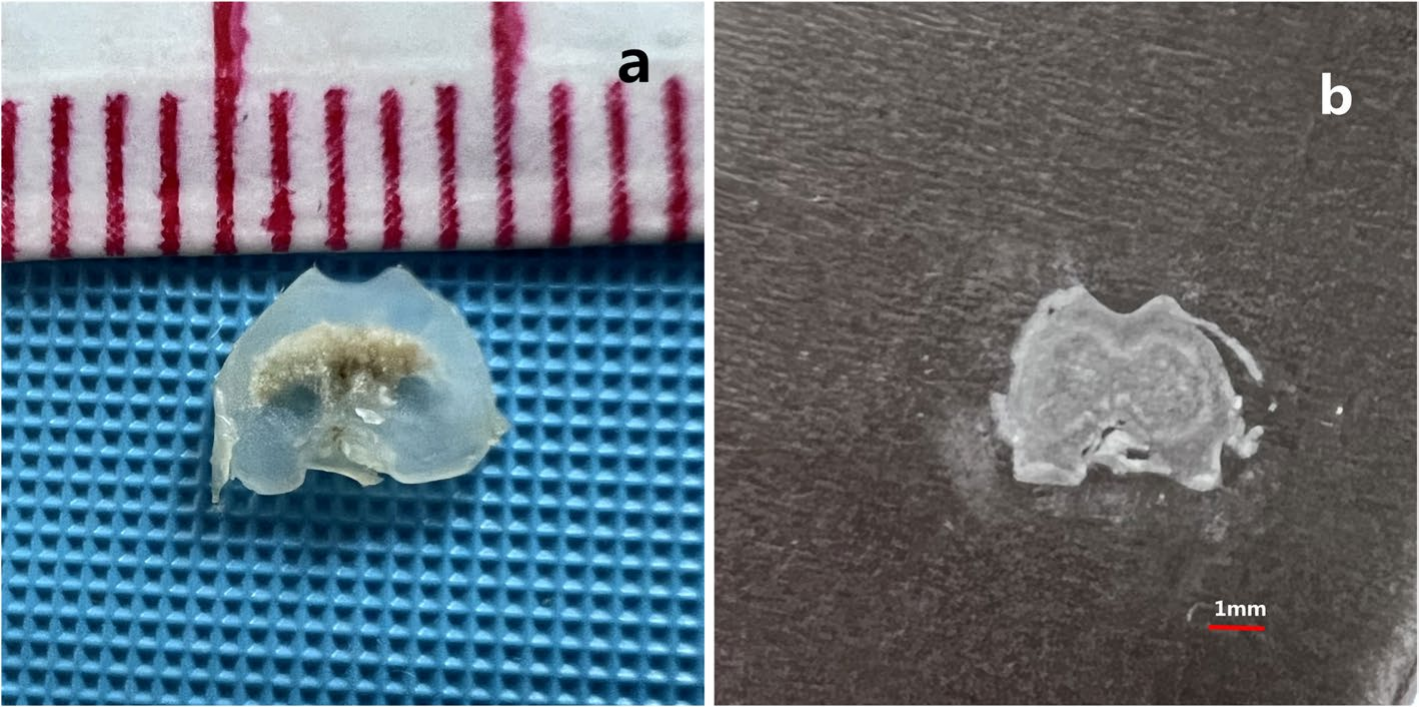
**a1** The transverse tissue section of the trochlear groove; **b1** The slice of the femoral trochlea before HE staining

**Fig. 3:**
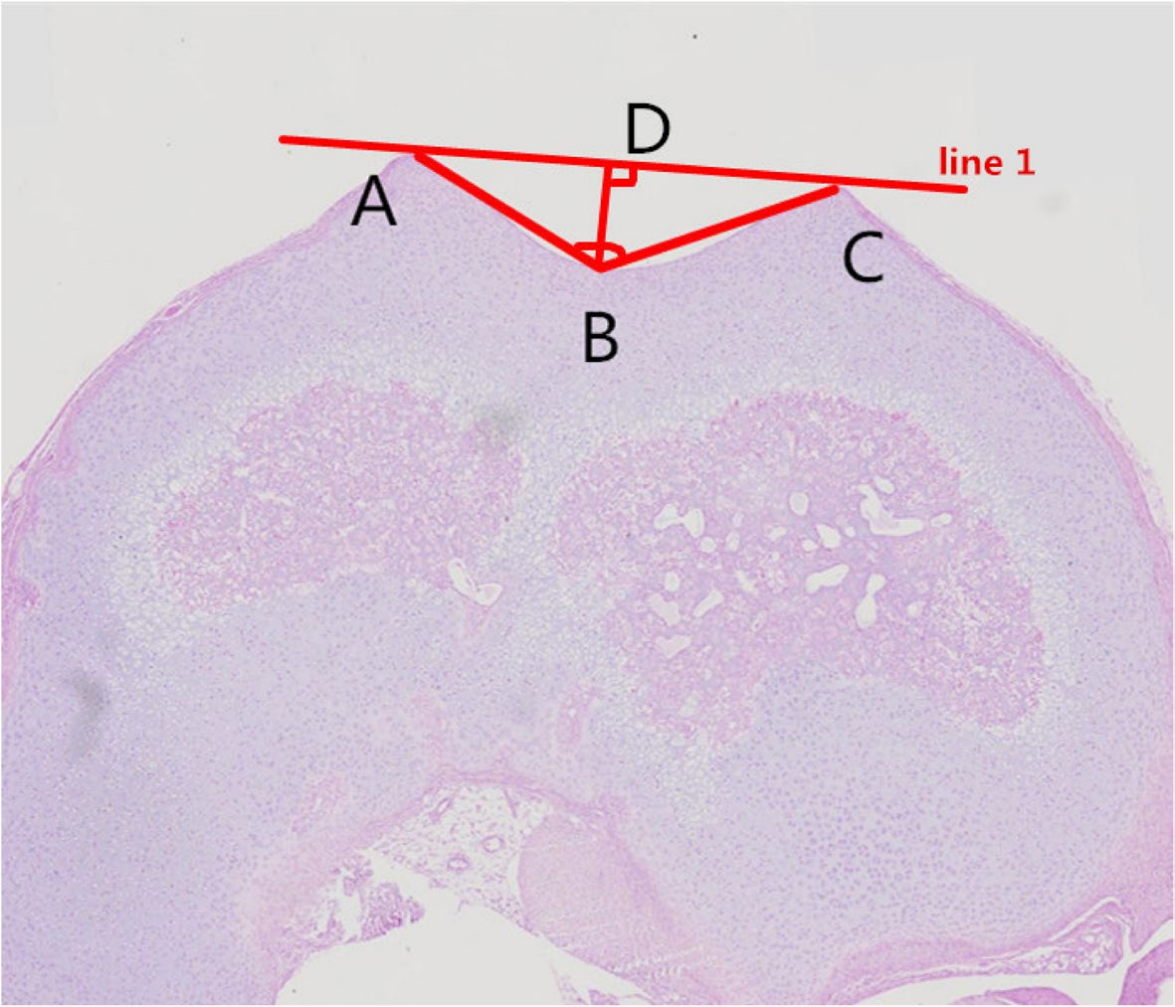
The angle ∠ABC is defined as the trochlear sulcus angle, AC is defined as the trochlear sulcus width; BD is defined as the trochlear sulcus depth

The specimens of knee were immersed in 4% paraformaldehyde (pH = 7.40) overnight at 4 °C and then transferred to 10% ethylene diamine tetraacetic acid (EDTA) solution for approximately 4-5 weeks for decalcification, followed by alcohol and xylenes gradient dehydration. The specimens were embedded in paraffin for subsequent tissue staining. Next, tissue slices were cut into 5-μm along the femoral axis to get the transverse images of the trochlear groove (Fig. 2). Then, the sections were stained with HE to show the cartilage and subchondral bone [18, 19], and microscopic measurement of the morphology of femoral trochlea was performed. The pictures of representative sections were recorded by a camera. The cartilaginous trochlear sulcus angle which was defined as the angle of the deepest point of the trochlear connecting with the lateral trochlear cartilaginous surface and the medial trochlear cartilaginous surface at the same slice. The methods applied to the measurement of cartilaginous trochlear sulcus angle, trochlear sulcus width and depth are summarized in Table 1 and Fig. 3. For determining the inter-observer variation, measurements were performed by two authors, who were blinded to the grouping. To determine the intra-observer variation, one author repeated the observations at 3 days after the first measurement.

**Table 1:** Description of measurements

Morphological characteristics of the trochlear groove	
Trochlear sulcus angle (∠ABC)	The angle between the slopes of the medial and lateral trochlear groove. (Fig. 2)
Trochlear sulcus depth (BD)	Line 1 is drawn across the trochlear groove of the medial and lateral condyles. Trochlear sulcus depth is the distance from the deepest portion (B) of the trochlear sulcus to line 1(D). (Fig. 2)
Trochlear sulcus width (AC)	The width is the distance from the lateral condyle (A) to the medial condyle (C). (Fig. 2)

### Statistical analysis

SPSS statistical software (version 22.0; SPSS, IL, USA) was performed for data analyses. All descriptive data values were expressed by mean±SD. The inter-and intraobserver reliabilities were then determined by calculating intra-class correlation coefficients (ICCs). The chi-square test was used to analyze differences between sexes. The normality of the distribution of values for each variable was assessed by the Shapiro–Wilks test and Dunnett’s multiple-comparisons was used for evaluation between each experimental group and control group at each time point. Independent-Samples T test was used for analysis of parametric data. P-value < 0.05 was defined as the threshold for statistical significance.

## Results

The sulcus angle had a measurement accuracy of 0.1° , and the trochlear sulcus width and depth had a measurement accuracy of 0.01mm. The inter-and intra-observer correlation coefficients were high between measurements (Table 2). All the rats in the three groups developed normally, and the weights of rats did not show significant differences at different stages of development.

**Table 2:** Intra-observer and inter-observer agreement of geometric measurements with 95% confidence intervals(CI)

Measurement	Intra-observer		Inter-observer	
	ICC	95% CI	ICC	95% CI
SSG-TSA	0.785	0.746 to 0.825	0.723	0.687 to 0.815
SSG-TSD	0.802	0.707 to 0.859	0.768	0.615 to 0.804
SSG-TSW	0.853	0.787 to 0.939	0.868	0.805 to 0.924
PSG-TSA	0.783	0.696 to 0.827	0.702	0.651 to 0.785
PSG-TSD	0.786	0.713 to 0.838	0.754	0.726 to 0.837
PSG-TSW	0.836	0.793 to 0.906	0.855	0.788 to 0.930
CG-TSA	0.863	0.814 to 0.906	0.828	0.735 to 0.908
CG-TSD	0.786	0.708 to 0.855	0.783	0.717 to 0.893
CG-TSW	0.827	0.735 to 0.886	0.850	0.803 to 0.936

*ICC* intra-class correlation coefficient, *SSG* short swaddling group, *TSA* trochlear sulcus angle, *TSD* trochlear sulcus depth, *TSW* trochlear sulcus width, *PSG* prolonged swaddling group, *CG* control group

### Gross observation

**Fig. 4:**
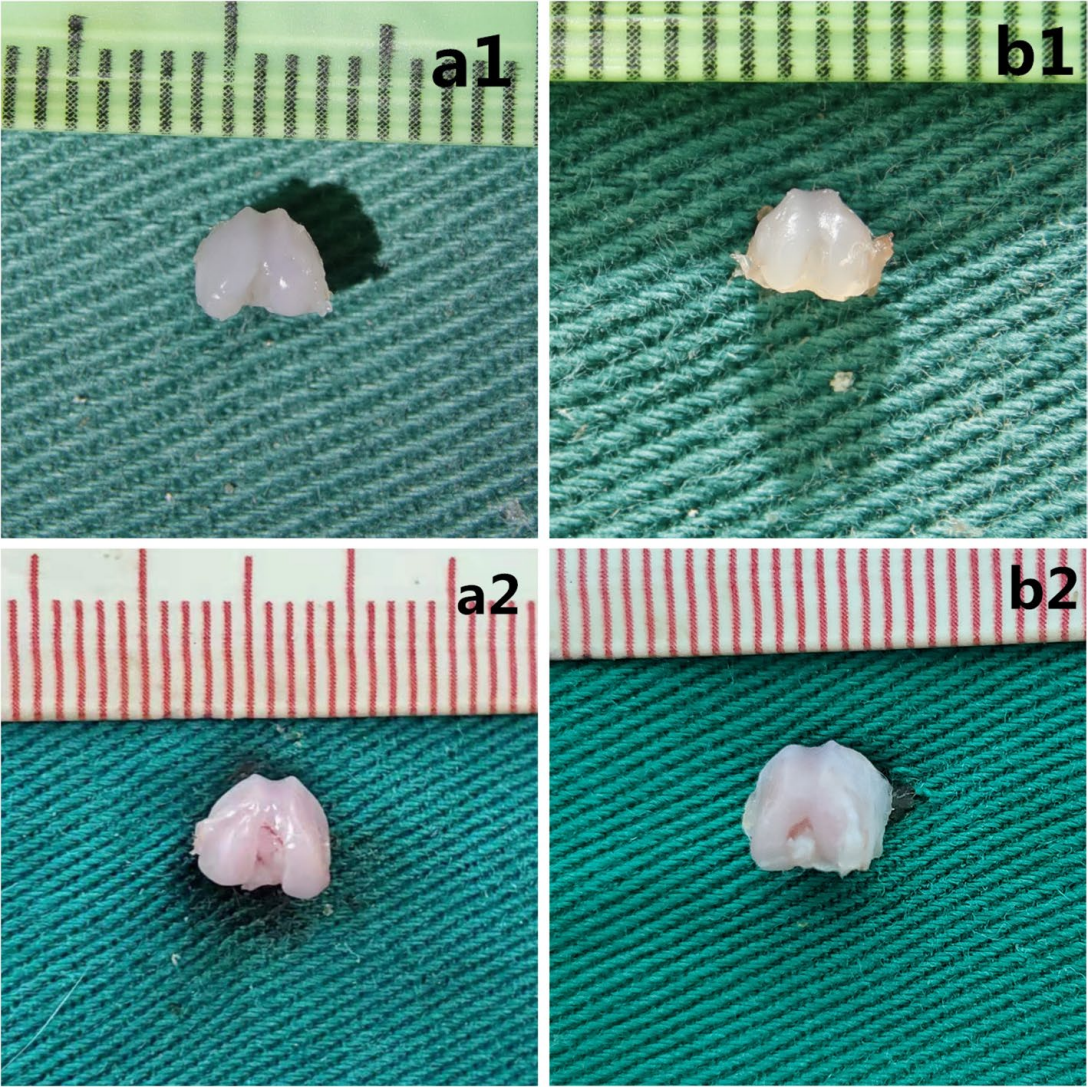
Gross anatomy of the femoral trochlea: **a1** 5 days of the control group; **b1** 5 days of the short Swaddling group; **a2** 10 days of the control group and **b2** 10 days of the prolonged Swaddling group

At 5 days after birth, the surface of the articular cartilage was smooth and the luster was bright in the short swaddling group and control group. However, compared to control group, the short swaddling group had flat trochlear grooves. At 10 days after birth, no significant difference was noted in the surface of the articular cartilage between the prolonged swaddling group and control group. The trochlear grooves were shallower in prolonged Swaddling group than in the control group (Fig. 4).

### Cross-sectional observation of trochlear morphology

**Fig. 5:**
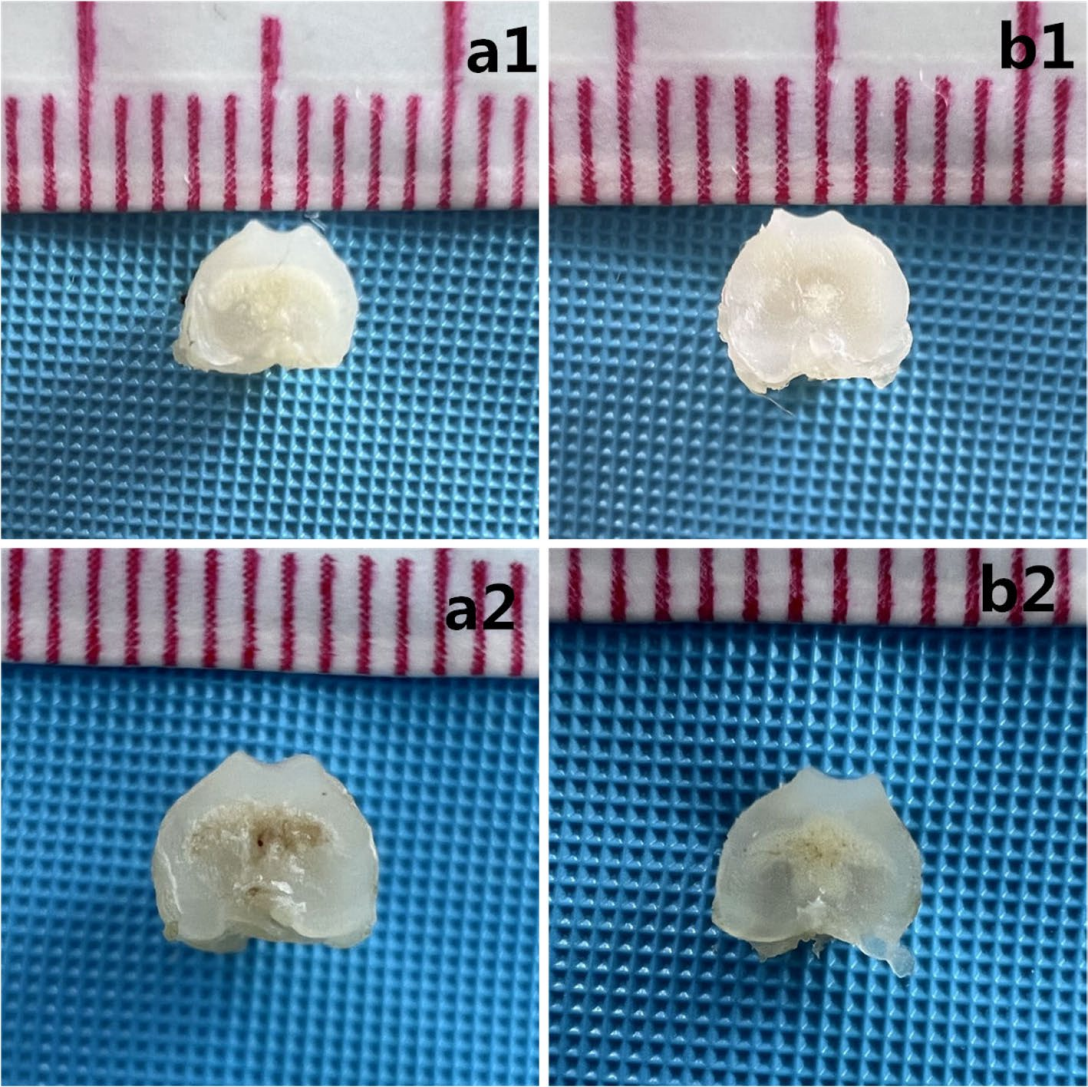
Cross-sectional observation: axial image of the femoral trochlea: **a1** 5 days of the control group; **b1** 5 days of the short Swaddling group; **a2** 10 days of the control group; **b2** 10 days of the prolonged Swaddling group

Compared to the control group, the short swaddling group had an increase in the trochlear sulcus angle and decrease in the depth of the trochlear grooves, which were shallow. At 10 days after birth, the difference of the trochlear groove between the prolonged swaddling group and control group was further aggravated. Compared to the control group, the trochlear groove was more flattened in the prolonged swaddling group (Fig. 5).

### Histological analysis

We defined the trochlear dysplasia of the two experimental groups if the trochlear sulcus angle had 5°greater than the average degree of the control group.

**Fig. 6:**
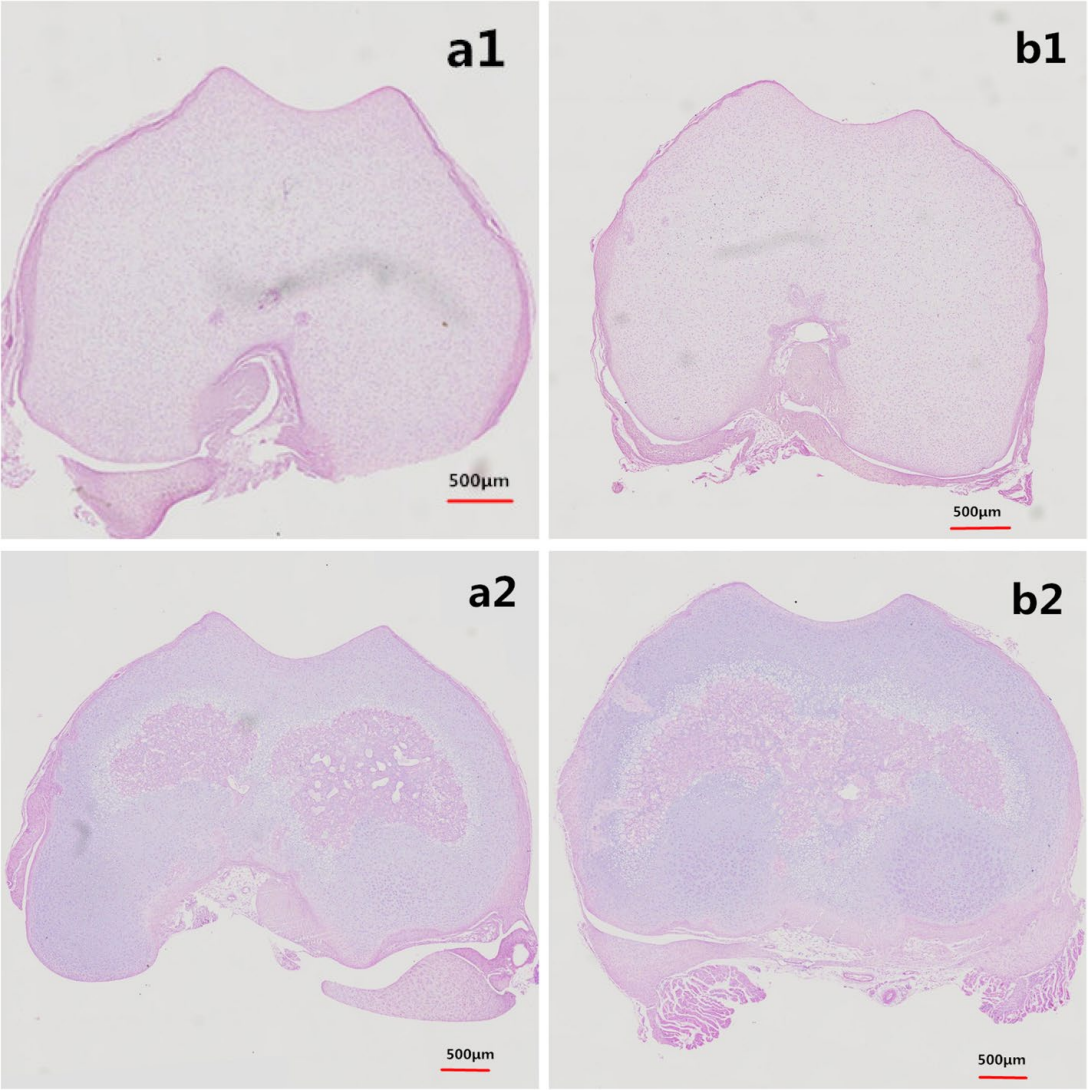
Microscopic view of femoral trochlear by HE staining: **a1** 5 days of the control group, **b1** 5 days of the short Swaddling group; **a2** 10 days of the control group and **b2** 10 days of the prolonged Swaddling group

It can be seen on the panorama of the pathological sections that the arrangement of the articular cartilage cells were orderly, and the surface of the articular cartilage was smooth in both short Swaddling group and control group at 5 days after birth. Compared with the control group, the trochlear groove was shallower in the short Swaddling group. At 10 days after birth, the difference of the trochlear groove between the prolonged Swaddling group and control group became more serious. However, the surface and arrangement of the articular cartilage cells were not obviously different between the two experiment groups and the control group (Fig. 6).

At 5 days after birth, the average degree of trochlear sulcus angle was significantly greater in the short Swaddling group than in the control group. The mean trochlear sulcus depth was shallower in the short Swaddling group than in the control group. There were significant differences between the short Swaddling group and control group at 5 days after birth (P < 0.01). No significant difference was seen in the trochlear sulcus width between the short Swaddling group and the control group at 5 days after birth (P = 0.56) (Table 3). Compared with the average degree, there were 42 trochleas from 21 rats (female 14/30, male 7/30) that developed trochlear dysplasia among the total 30 rats of the short Swaddling group.

**Table 3:** The measurements of trochlear sulcus of control group and short swaddling group (X ± SD)

Indexes group	Short swaddling	Control group	*P*-value^a^
Mean TSA°	136.5±4.9	130.2±3.4	<0.01
Mean TSD(mm)	0.23 ± 0.04	0.31 ± 0.03	<0.01
Mean TSW(mm)	1.28 ± 0.02	1.27 ± 0.02	0.56

^a^Student’s t-test

*TSA* trochlear sulcus angle, *TSD* trochlear sulcus depth, *TSW* trochlear sulcus width

At 10 days after birth, the average degree of trochlear sulcus angle in the prolonged Swaddling group was significantly different from that in the control group (P < 0.01). The mean trochlear sulcus depth was significantly shallower in the prolonged Swaddling group than in the control group (P < 0.01). However, the mean trochlear sulcus width was not found to be significantly different between the prolonged Swaddling group and the control group at 10 days after birth (P = 0.53) (Table 4). There were 52 trochleas from 26 rats (female 14/30, male 12/30) that developed trochlear dysplasia.

**Table 4:** The measurements of trochlear sulcus of control group and prolonged swaddling group (X ± SD)

Indexes	Prolonged swaddling group	Control group	*P*-value^a^
Mean TSA°	135.8±4.6	128.5±3.4	<0.01
Mean TSD(mm)	0.32 ± 0.03	0.42 ± 0.02	<0.01
Mean TSW(mm)	1.53 ± 0.02	1. 52 ± 0.02	0.53

^a^Student’s t-test

*TSA* trochlear sulcus angle, *TSD* trochlear sulcus depth, *TSW* trochlear sulcus width

Although there was a greater number of female rats than male rats, the trochlear dysplasia appeared in the different experimental group, and the differences between sexes were not significant (Table 5).

**Table 5:** Demographic characteristics of the short swaddling group and prolonged swaddling group

	Short swaddling group (n = 30)	Prolonged swaddling group (n = 30)	Chi-Square	P
Gender				
Female	14/30	14/30	0.157	3.84
Male	7/30	12/30		

## Discussion

The key findings of the current study were that breech presentation could cause trochlear dysplasia in neonatal rats and that the incidence of trochlear dysplasia increased with the Swaddling time.

Trochlear dysplasia that is characterized by a shallow, flattened trochlear groove is one of the most common knee disorders in children and adolescents with an incidence of 29 to 43 per 100 000 [20]. Currently, there has been no consensus on the accurate aetiology of trochlear dysplasia and the related risk factors. However, the numerous evidence in the previous studies has revealed the correlation of trochlear dysplasia with the risk factors [21]. Breech presentation has long been reported to be a potential risk factor for trochlear dysplasia [10, 11, 22]. It is well known that the cartilaginous trochlear develops early on in gestation, between 9 and 16 weeks [23]. Given that trochlea appears well established in the prenatal period, it is conceivable that an intrauterine developmental process may be the aetiology of trochlear dysplasia [24].

The relationship between breech presentation and trochlear dysplasia had been demonstrated through epidemiological methods [11, 24]. DeVries CA et al. [24] recently reported that the incidence of trochlear dysplasia in breech presentation was 13.5%. Patients with breech presentation were found to have a shallower and flatter trochlear groove than those with-not breech presentation [24]. The study by Øye CR reported that knees extended position could bring about a 45-fold increased risk of dysplasia compared to knees with free to flex [11]. Although the relationship between breech presentation and trochlear dysplasia have been well demonstrated [11, 24], there have been no experimental studies indicating the pathological process of breech position-related trochlear dysplasia. Therefore, the study aimed at disclosing the pathogenesis of breech-related trochlear dysplasia based on a neonatal rat model.

Immature animal models have been frequently used to study the risk factors for trochlear dysplasia [21, 25, 26]. In the study, the rats in hip flexion and knee extension were fixed with medical tape to simulate the intrauterine breech posture that was most associated with trochlear dysplasia [11, 12]. Compared to rigid fixation, the elasticity of medical tape could allow for minor movement which was closer to the natural circumstance of intrauterine breech position [12]. It is known that breech presentation was fixed by the time of 32 weeks of gestation [27]. In general, approximately 85% ~ 90% of children born in the breech position had the knees extended [28]. Therefore, we used swaddling right similar to that in human breech presentation after birth in order that the developmental potential and morphology of the knee was the closest to intrauterine status.

In the study, a neonatal rat model was established to simulate the intrauterine breech posture. At 5 days after birth, the average degree of trochlear sulcus angle was significantly greater in the short Swaddling group than in the control group (*P* < 0.01). The mean trochlear sulcus depth was shallower in the short Swaddling group than in the control group (*P* < 0.01). However, no significant difference was seen in the trochlear sulcus width between the short Swaddling group and the control group at 5 days after birth (*P* = 0.56). Compared with the average degree, trochlear dysplasia was detected in 42 trochleas from 21 rats (female 14/30, male 7/30) among the total 30 rats of the short Swaddling group. At 10 days after birth, the average degree of trochlear sulcus angle in the prolonged Swaddling group was significantly different from that in the control group (*P* < 0.01). The mean trochlear sulcus depth was significantly shallower in the prolonged Swaddling group than in the control group (*P* < 0.01). The mean trochlear sulcus width were not found to be significantly different between the prolonged Swaddling group and the control group at 10 days after birth (*P* = 0.53). Trochlear dysplasia developed in 52 trochleas from 26 rats (female 14/30, male 12/30). This indicated that breech presentation was associated with trochlear dysplasia, and the pathogenesis of breech-related trochlear dysplasia was a progressive process. In the study, prolonged swaddling caused a 16.7% increase in the prevalence of trochlear dysplasia (from 70% after short swaddling for 5 days to 86.7% after prolonged swaddling for 10 days). This finding suggests that the severity of the pathological changes increases as the knees remain under this condition for a longer period.

It is universally acknowledged that there is unique matching relation of the patella and femoral trochlea, which is the basis of its biomechanical function [29]. Stress stimulation plays an important role in articular cartilage and subchondral bone development [7]. Researches have shown that mechanical stress is one of the most important factors affecting the cartilage and bone development [30, 31]. During knee bending, the patella enters the femoral trochlear groove track and generates mechanical stress between the femoral trochlea and the patella. The mechanical loading from the patella is transmitted from the trochlear cartilage to subchondral bone. This mechanical stress stimulates the growth and remodelling of the femoral trochlea and the patella [6]. DeVries CA et al. [24] found that breech positioning was associated with trochlear dysplasia, which is consistent with our finding. It is known that the patella is not seated in the trochlear groove until 30° of knee flexion. So we believe that the main reason for trochlear dysplasia is the reduction of mechanical stress in the patella. In the study, no significant difference was found with respect to the mean trochlear sulcus width between the control group and each experimental group. The findings indicate that mechanical stress does not stimulate the growth of trochlea sulcus width in a short period after breech presentation. The female gender had been recognized as a risk factor for trochlear dysplasia. There was a female: male ratio of greater than 3:1 [3, 32]. In our study, the prevalence of trochlear dysplasia was higher in female rats than in male rats in the short swaddling group. However, no significant difference was noted in the incidence of trochlear dysplasia between the two genders in the prolonged Swaddling group. The application of swaddling might have a more overwhelming effect than that of sexual differences. The overwhelming effect of swaddling were also observed in previous studies [12, 33].

This study has several limitations. First, we used neonatal rats to simulate breech presentation rather than establishing a real intrauterine animal model, which made it difficult to completely reflect the anatomical situation of the human. Ren et al. [12] had endeavored to establish such a simulate breech presentation model by connecting the hindlimb skin with the chest wall through intrauterine operation. However, none of the operated pups survived the delivery. Second, a continuous observation of the knee joint in animal model would have been more conclusive. However, radiological methods such as X-ray or computerized tomography (CT) could not function well in such tiny knees in neonatal rats. Third, the study included the investigation of femoral groove development. However, further studies to include the morphological changes of patella could also be performed in order to get more information on breech presentation.

## Conclusions

In conclusion, to our knowledge, the present study was the first to investigate the pathological process of breech-related trochlear dysplasia. Breech presentation had an adverse effect on neonatal knees and could induce trochlear dysplasia. There was a positive linear correlation between swaddling time and the incidence of trochlear dysplasia. Breech-related trochlear dysplasia was a chronic process that proceeded from shallower trochlear groove to trochlear dysplasia.

## Data Availability

The detailed data and materials of this study were available from the corresponding author through emails on reasonable request.

## Abbreviations

SSG: Short swaddling group
PSG: Prolonged swaddling group
CG: Control group
HE: Haematoxylin and eosin
EDTA: Ethylene diamine tetraacetic acid
ICCs: Intra-class correlation coefficients
CT: Computerized tomography
